# Atomic layer deposition and properties of ZrO_2_/Fe_2_O_3_ thin films

**DOI:** 10.3762/bjnano.9.14

**Published:** 2018-01-10

**Authors:** Kristjan Kalam, Helina Seemen, Peeter Ritslaid, Mihkel Rähn, Aile Tamm, Kaupo Kukli, Aarne Kasikov, Joosep Link, Raivo Stern, Salvador Dueñas, Helena Castán, Héctor García

**Affiliations:** 1 Institute of Physics, University of Tartu, W. Ostwald 1, 50411 Tartu, Estonia; 2 Department of Chemistry, University of Helsinki, P. O. Box 55, FI-00014 Helsinki, Finland; 3 National Institute of Chemical Physics and Biophysics, Akadeemia tee 23, 12618 Tallinn, Estonia; 4 Department of Electronics, University of Valladolid, Paseo Belén 15, 47011 Valladolid, Spain

**Keywords:** atomic layer deposition, metal oxides, thin films

## Abstract

Thin solid films consisting of ZrO_2_ and Fe_2_O_3_ were grown by atomic layer deposition (ALD) at 400 °C. Metastable phases of ZrO_2_ were stabilized by Fe_2_O_3_ doping. The number of alternating ZrO_2_ and Fe_2_O_3_ deposition cycles were varied in order to achieve films with different cation ratios. The influence of annealing on the composition and structure of the thin films was investigated. Additionally, the influence of composition and structure on electrical and magnetic properties was studied. Several samples exhibited a measurable saturation magnetization and most of the samples exhibited a charge polarization. Both phenomena were observed in the sample with a Zr/Fe atomic ratio of 2.0.

## Introduction

Doped ZrO_2_ has been a subject of interest because of several potential applications, for example, in microelectronics as a memory material [1]. Also, doping a dielectric film with a magnetic material might provide the structural distortion required to stabilize the ferroelectric phase thus resulting in a multiferroic material, which would allow an additional degree of freedom in device design [2].

ZrO_2_ doped with various chemical elements has been studied for several applications and different processes have been employed to prepare the samples. Ca- and Mg-stabilized cubic zirconia, prepared by pulsed laser deposition (PLD), has shown ferromagnetic properties [3]. Magnetic properties of PLD-synthesized ZrO_2_, doped with Co, Fe, Mn or Ni, have been studied [4], showing that doping ZrO_2_ with Mn results in a significantly higher saturation magnetization than doping ZrO_2_ with the other transition metals studied. Fe–ZrO_2_ nanocomposite thin films have been synthesized using a solid state reaction between the Zr and Fe_2_O_3_ layers, and their composition, structure, chemical stability and magnetic properties were characterized [5]. Upon annealing at 500 °C, the metastable cubic phase of ZrO_2_ was stabilized and ferromagnetic hysteresis of the nanocomposite film was confirmed. Saturation magnetization was measured to be ≈173 emu/g [5]. Undoped ZrO_2_, prepared by pulsed electron beam deposition [6] or reactive DC magnetron sputtering [7], also exhibited ferromagnetic properties. The undoped ZrO_2_ exhibited ferromagnetic properties mainly driven by oxygen vacancies. Monoclinic and tetragonal phases with similar amounts of oxygen vacancies were compared and ferromagnetism was only observed in the case of the tetragonal phase [7]. Microwave-assisted combustion synthesis of a powder and the subsequent sintering of samples were used to fabricate ZrO_2_ doped with Co, and ferromagnetism in such samples was confirmed [8]. Different nanostructures of undoped ZrO_2_ were prepared by catalyst-assisted PLD and all structures were found to possess ferromagnetic behavior [9]. Ferromagnetism was also observed in annealed Co and Fe co-doped ZrO_2_, prepared by the sol–gel method [10]. Samples of Mn- and Fe-stabilized cubic zirconia were obtained by a co-precipitation method and no ferromagnetism was observed in such samples [11]. Phase diagrams for the ZrO_2_–FeO system were described [12] and the inﬂuence of thermal treatment on the phase development in ZrO_2_–Fe_2_O_3_ and HfO_2_–Fe_2_O_3_ systems was assessed [13].

ALD of ZrO_2_ from ZrCl_4_ and O_3_ has been studied [14]. Reactions between Fe(acac)_3_ adsorbing on zirconia surfaces [15–16] has been studied as well. Phase stabilization of ZrO_2_ by Fe doping was investigated by using ALD [17]. Ferromagnetism in ALD-grown Fe_3_O_4_/ZrO_2_/Fe_3_O_4_ multilayer nanotubes has been demonstrated [18], while the precursors for distinct solid oxide layers constituting these samples were ferrocene/ozone and tetrakis(dimethylamido)zirconium(IV)/water for Fe_2_O_3_ and ZrO_2_, respectively, and Fe_2_O_3_ was reduced to Fe_3_O_4_ after the growth. In another study [19], ZrO_2_/Fe thin films were prepared by ALD from β-diketonate precursors and ozone. After annealing at 600 °C in N_2_ flux for 60 s, the films exhibited ferromagnetic properties. The ferroelectric properties of ALD-grown undoped zirconia have also been investigated [20]. ZrO_2_ thin films were deposited by remote plasma ALD from tetrakis(dimethylamido)zirconium(IV) and oxygen plasma, and were found to exhibit ferroelectric behavior. ALD and physical vapor deposited ZrO_2_ were compared from the viewpoint of ferroelectric behavior [21], whereby ALD precursors were Zr-based metal organic precursors (TEMAZr) and H_2_O. Both fabrication methods provided samples with antiferroelectric behavior.

Here, ZrO_2_ films doped with Fe_2_O_3_ were grown by atomic layer deposition from zirconium chloride and ferrocene precursors. The purpose of this study was to investigate the effects of the Zr/Fe cation ratio on the film structure as well as on the magnetic and electrical properties, and to examine whether alternately deposited iron and zirconium oxides form dilute solids or become segregated upon thermal deposition and processing. The goal of the magnetic and electrical measurements was to evaluate the ability of ZrO_2_/Fe_2_O_3_ films to polarize in both electric and magnetic fields and thus clarify to what extent may such materials exhibit multiferroic behavior.

## Experimental

The ZrO_2_/Fe_2_O_3_ films were grown in a low-pressure (200–260 Pa) flow-type in-house built hot-wall ALD reactor [22] at 400 °C. Zirconium tetrachloride, ZrCl_4_ (Aldrich, 99.99%), and ferrocene, Fe(C_5_H_5_)_2_ (ABCR, 99%), were used as zirconium and iron precursors, respectively. Ozone, O_3_, was used as the oxidizer. Nitrogen, N_2_ (99.999% purity, AGA), was applied as the carrier and purging gas. At the temperature chosen (400 °C) the ZrO_2_ grows efficiently from ZrCl_4_ and O_3_ [14], and this temperature is also sufficiently high to ensure efficient growth also for iron oxide from cyclopentadienyls and ozone [23]. ALD growth of Fe_2_O_3_ from ferrocene and ozone is even possible at 200 °C, but at such a low temperature, the required pulse duration is very long. A maximal growth rate was achieved when ferrocene pulses were 40 s long and ozone pulses were 200 s [24]. ZrCl_4_ and Fe(C_5_H_5_)_2_ were evaporated at 161–163 °C and 61–63 °C, respectively, from open boats inside the reactor and transported to the substrates by the carrier gas ﬂow.

Ozone was produced from O_2_ (99.999% purity, AGA) using a BMT Messtechnik 802 N generator. The ozone concentration, measured using a BMT Messtechnik 964 analyzer, was 245–250 g/m^3^ in the experiments, which is 17.1–17.5%. The estimated ozone flow rate from the generator was about 67 sccm, while the carrier gas flow rate was kept at about 220 sccm.

The ZrO_2_/Fe_2_O_3_ films were grown by alternately applying certain amounts of constituent binary oxide growth cycles. The ZrO_2_/Fe_2_O_3_ cycle ratio was varied as 10:1, 10:3, 10:5, 10:10, and 5:5. The cycle times used for both ZrO_2_ and Fe_2_O_3_ were 5–5–5–5 s for the sequence of metal precursor pulse–purge–O_3_ pulse–purge. Each deposition started and ended with pulses of ZrO_2_ in order to make the film symmetrical from electrode to electrode in terms of the chemical composition. Altogether, 220–235 ALD cycles were deposited to obtain each thin film.

The films were grown on various substrates: Si(100) and highly doped conductive Si substrates covered by a 10 nm TiN film grown by chemical vapor deposition. Before deposition, the Si(100) was rinsed in a mixture of H_2_SO_4_/H_2_O_2_ 5:2 and heated on a hot plate at 80 °C. After that, the Si(100) was cleaned with distilled water in an ultrasound container. The next step was to clean the samples with a 7% solution of HF and again clean with distilled water under ultrasound. During the time after cleaning and before placing the samples in a reactor, a SiO_2_ layer of a few nanometers thick grew on the sample because of contact with surrounding air.

Selected samples on Si(100) were annealed at 850 °C and 1000 °C in air for 15 and 10 minutes, respectively. For electrical measurements, the films deposited on TiN substrates were supplied with platinum electrodes, with an area of 0.204 mm^2^, that were electron-beam-evaporated on top of the films. In addition, for investigating deposition uniformity in terms of conformal growth, stacked three-dimensional structures of silicon substrates with an aspect ratio of 1:20 were used. Such three-dimensional trenched or stacked structures are similar to those used in micro- and nanoelectronics as capacitor bottom electodes, allowing the exploitation of capacitor arrays with enlarged memory density [25–27].

The crystal structure was evaluated by grazing incidence X-ray diffractometry (XRD), using an X-ray diffractometer (SmartLab, Rigaku) using Cu Kα radiation, which corresponds to an X-ray wavelength of 0.15406 nm. A spectroscopic ellipsometer (model GES5-E) was used for the evaluation of the film thickness. The Tauc–Lorentz model was used for the modelling of samples in the range of 300–1000 nm. An X-ray fluorescence (XRF) spectrometer (Rigaku, ZSX 400) with the software program ZSX (version 5.55) was used to evaluate the elemental composition of the films. The surface morphology of the films and a cross-section of an ALD coated stack were evaluated by scanning electron microscopy (SEM) using a dual beam SEM (FEI, Helios NanoLab 600). An FEI Titan Themis 200 device was used for transmission electron microscopy (TEM) imaging of selected samples.

The electrical measurements were performed by means of an Agilent DXO-X 3104 digital oscilloscope with a built-in wave generator. The standard Sawyer–Tower experiment was carried out by applying a periodic triangular-shaped stimulus and recording the voltage loop data from the oscilloscope. The charge values were obtained from the sensed voltage across a stated capacitance.

Magnetic measurements were performed using a vibrating sample magnetometer (VSM) option of the physical property measurement system (14T, Quantum Design) by scanning the magnetic field from −1 T to 1 T parallel to the film surface at room temperature.

## Results and Discussion

### Film growth and composition

The film thickness varied between 15 and 40 nm. Regarding the composition, the cation ratio of mixed films in the as-deposited state, measured by X-ray fluorescence spectrometry, varied from Zr/Fe 0.15–50. Table 1 gives a list of the samples with a description of the growth cycle sequences as well as the observed range of composition in terms of the constituent metal ratio. Possibly, due to the different adsorption and nucleation rates of different metal precursors, the marked deviations in the chemical composition could not be avoided. One can also see that the oxide cycle ratio does not directly match with the cation ratio. This was actually expected because the nucleation of every oxide is slower with the growth rate retarded during the first few deposition cycles, and the content of metal deposited does not linearly correspond to the amount of additive cycles at the early stages of the growth. The nonlinearity of the thickness as a function of growth per cycle was also observed with HfO_2_ [28] and ZrO_2_ [29].

**Table 1:** List of the ZrO_2_/Fe_2_O_3_ films, with constituent oxide cycle ratios and full growth cycle sequences indicated, deposited on Si substrates (subjected to magnetometry) and TiN substrates (subjected to electrical measurements). The range of cation ratios is due to the variation of the ratios measured at different locations on the substrate holder and are due to the film thickness growth rate profiles along the gas flow direction. In these experiments, lower cation ratios were measured on TiN substrates as compared to Si substrates.

Cycle ratio	Cycle sequence	Zr/Fe cation ratio	Thickness on Si	Thickness on TiN

10:3	17 × [10 × ZrO_2_ + 3 × Fe_2_O_3_] + 10 × ZrO_2_	10	23 nm	21 nm
10:5	15 × [10 × ZrO_2_ + 5 × Fe_2_O_3_] + 10 × ZrO_2_	0.34–2.0	22 nm	15 nm
10:10	11 × [10 × ZrO_2_ + 10 × Fe_2_O_3_] + 10 × ZrO_2_	0.21–1.7	26 nm	19 nm
5:5	22 × [5 × ZrO_2_ + 5 × Fe_2_O_3_] + 5 × ZrO_2_	0.14–0.16	21 nm	36 nm

**Figure 1:**
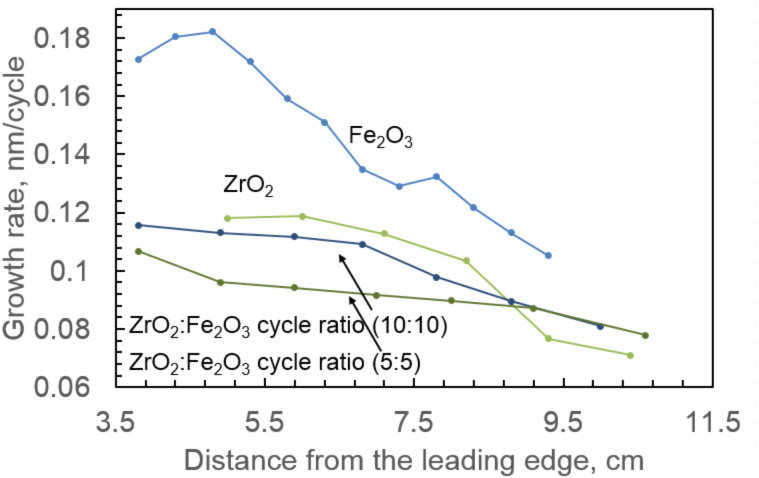
Growth rates of Fe_2_O_3_, ZrO_2_ and their mixtures with different cycle ratios as a function of sample position in the ALD reactor (distance between the sample and valve). All samples of mixed oxides exhibited lower growth rates than pure Fe_2_O_3_ or ZrO_2_. Mixtures with cycle ratios not shown in the image exhibited growth rates very similar to the shown mixtures.

The growth rate for a single deposition was different for each sample in the reactor because of the reactor type and positioning of the samples (Figure 1). The growth rate decreased with increasing distance between the sample and the valve, which releases precursors into the reactor. Pure Fe_2_O_3_ exhibited the highest growth rate of 0.18 nm/cycle at a position, which was closest to the precursor valve. The growth rate of ZrO_2_ was 0.12 nm/cycle at the same position. Mixtures of these oxides had lower growth rates than individual Fe_2_O_3_ or ZrO_2_, varying around 0.1 nm/cycle (Figure 1).

The thickness (growth rate) profiles along the gas flow direction may be regarded as characteristic of cross-flow atomic layer deposition reactors, in which the substrate holder is designed in a way that leaves one edge of the substrate pointing towards the gas flow inlet and the other, trailing, edge closer to the pumping line. In the case of such arrangement, the gas flow in the reactor chamber is parallel to the surface plane from one fixed point towards another. Although the precursor pulses are separated by purge periods, the separation is not perfect during the actual operation. The adsorption waves may partially overlap and meet in the vicinity of the substrate surface, causing the chemical vapor deposition to be less controlled. At the leading edge, the film thickness is usually higher and gradually decreases towards the trailing edge. One can recognize this phenomenon on the basis of the curves in Figure 1. The extent of the profile depends on the reactivity of the precursors, process temperature, and pulse time parameters, and has been experimentally observed, for example, in an early study devoted to ALD of ZrO_2_ from ZrCl_2_ and H_2_O [30] and later in the case of ALD of ZrO_2_ from ZrCl_4_ and O_3_ [14]. This was generalized in a more theoretical study on the adsorption process during ALD [31]. Such profiles are likely and expected to be different for each constituent material.

Although the thickness and composition were not constant during a single deposition in the present study, all comparisons between different depositions have been made using samples that were at the same location in the reactor (i.e., that have the same distance from the leading edge).

The analysis of the light residue in the host materials similar to those grown in the present study has been described earlier in terms of the most critical residue components, i.e., chlorine and hydrogen [14]. The time-of-flight elastic recoil detection analysis veriﬁed quite low residual chlorine content with an average amount of 0.14 ± 0.01 atom % in the ﬁlms grown at 300 °C. Similar, rather low amounts were found in the films grown at 400 °C. The amount of hydrogen was 0.30 ± 0.03 atom % in the ﬁlm grown at 300 °C, and decreased to 0.14 ± 0.03 atom % in the ﬁlm grown at 400 °C.

### Film structure

**Figure 2:**
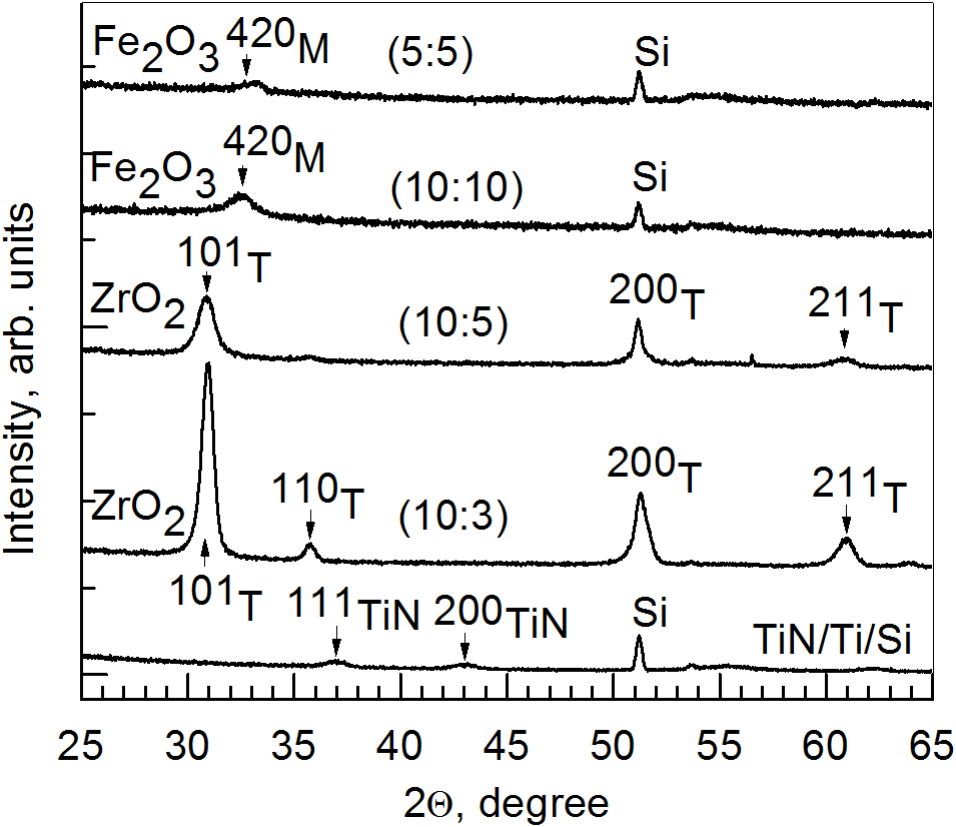
Grazing incidence X-ray diffraction (XRD) patterns for ZrO_2_/Fe_2_O_3_ films deposited on TiN with ZrO_2_/Fe_2_O_3_ cycle ratios and thickness indicated in the labels. The Miller indices are attributed to corresponding monoclinic (M) and tetragonal (T) phases of pure Fe_2_O_3_ and ZrO_2_, respectively. The cation ratio of Zr/Fe in the films deposited on TiN with cycle ratios of 5:5, 10:10, 10:5 and 10:3 were 0.15, 1.7, 2.0 and 10, respectively.

**Figure 3:**
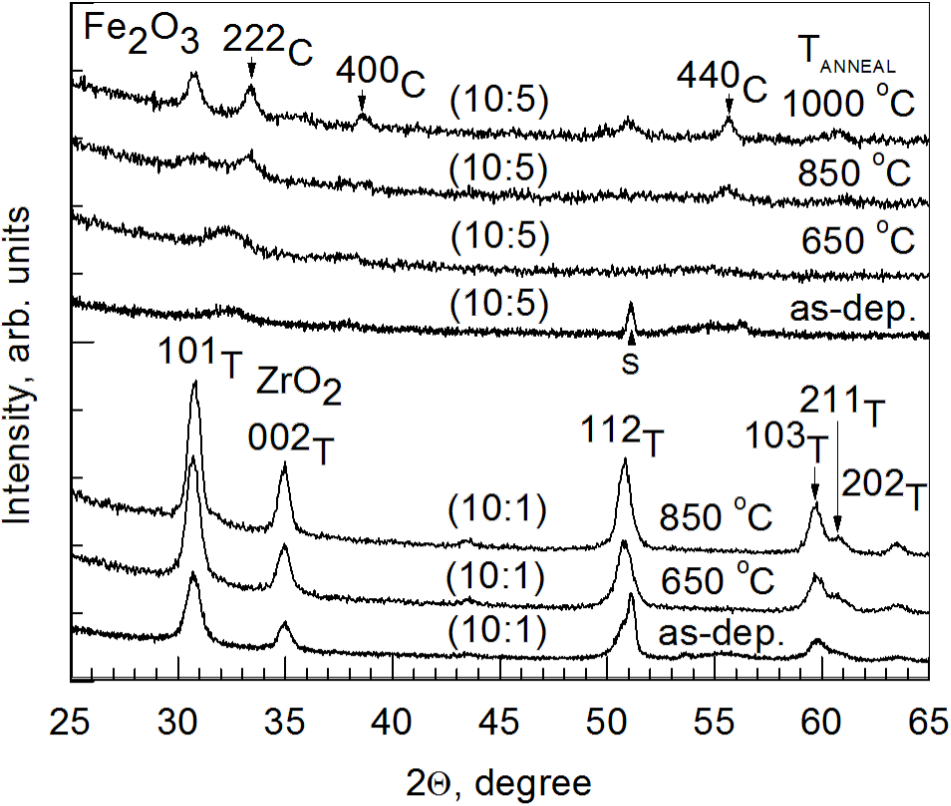
Grazing incidence X-ray diffraction (XRD) patterns for ZrO_2_/Fe_2_O_3_ films deposited on Si(100) with ZrO_2_/Fe_2_O_3_ cycle ratios and annealing temperatures indicated in the labels. The Miller indices are attributed to corresponding tetragonal (T) and cubic (C) phases of pure ZrO_2_ and Fe_2_O_3_, respectively.

It was shown that doping ZrO_2_ with Fe_2_O_3_ stabilized the tetragonal/cubic phase (PDF Card 70-6628) of ZrO_2_ for all substrates as long as the cation ratio of Zr/Fe exceed 2.0 (Figure 2 and Figure 3).

Lamperti et al. also determined the phase stabilization of ZrO_2_ by Fe doping [17] or otherwise in an ozone-based ALD of ZrO_2_ [32]. The stabilization of the tetragonal/cubic phase in Fe-doped ZrO_2_ has been confirmed by de Souza et al. [33] for samples fabricated by freeze-drying process and Kuryliszyn-Kudelska et al. [34] for samples prepared via wet chemistry. For the samples grown on TiN substrates, films with a higher Fe_2_O_3_ concentration did not show peaks assignable to any zirconia phase, but demonstrated reflections from crystallized Fe_2_O_3_ in the monoclinic phase (PDF Card 016-0653) (Figure 2). Ternary iron–zirconium oxide phases were not recognized. It is worth noting that, despite few existing works indicating the target material with the stoichiometry of ZrFe_2_O_5_, the synthesis of ZrFe_2_O_5_ has not been convincingly completed. Instead, one can believe that the solubility of iron oxide in zirconia is rather low and, in addition, it requires rather aggressive heat treatments. No crystallographic traces of ternary ZrFe_2_O_5_ have been registered, and distinct phases of Fe_2_O_3_ and ZrO_2_ have also earlier determined on the basis of the XRD patterns [13,35]. In our study, also alternative, zirconium-rich, ternary phases Zr_2_FeO*_x_* (PDF Card 019-0646), Zr_4_Fe_2_O_0.6_ (PDF Card 038-1168), and Zr_6_Fe_3_O (PDF Card 017-0559) were not recognized in XRD patterns. Samples on TiN and Si(100) substrates were different in their composition due to their different position in the reactor and the effect of a certain lateral composition profile. This is probably caused by differences in the adsorption rates of the precursors and concurrently different growth rate profiles (Figure 1) due to the appearance of the thickness profiles as described above.

Annealing the samples of lightly doped zirconia caused the grain growth in metastable tetragonal/cubic phase (Figure 3). Annealing samples with a ZrO_2_/Fe_2_O_3_ cycle ratio (10:1) and Zr/Fe = 50 resulted in the increased crystallization of the tetragonal/cubic phase of ZrO_2_ (Figure 3). This was also observed in the case of Zr/Fe = 10 (not shown). A similar result was also obtained by Štefanić et al. [13] who also showed that lightly Fe-doped ZrO_2_ crystallized in tetragonal/cubic zirconia phase after annealing. In the case of Zr/Fe = 0.34 and a cycle ratio of 10:5, the film was almost amorphous in the as-deposited state, but the crystallization of tetragonal/cubic ZrO_2_ and cubic Fe_2_O_3_ (PDF Card 04-0755) was observed upon annealing at 1000 °C in air for 10 min (Figure 3).

**Figure 4:**
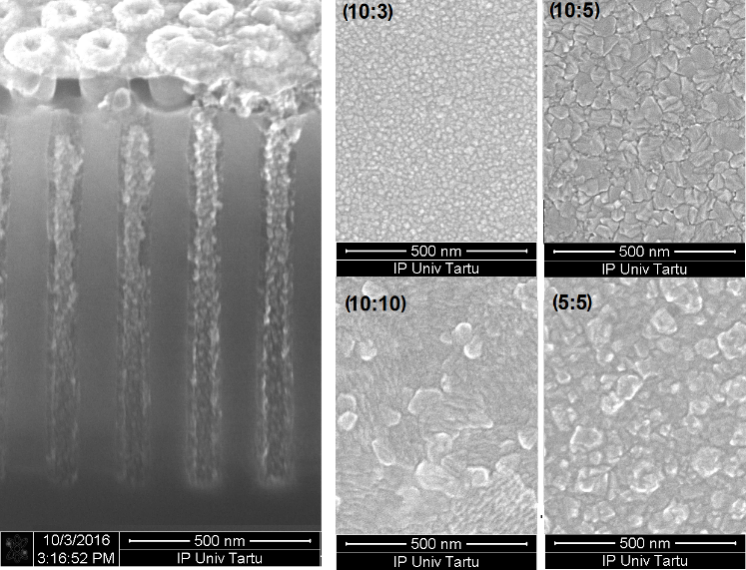
SEM image of a ZrO_2_/Fe_2_O_3_ film grown in a 3D stacked structure (left panel) and images of ZrO_2_/Fe_2_O_3_ film surfaces (right panel). The cycle ratios of the respective films are shown on each image of the right panel.

Since the ultimate goal of any materials synthesis process are its application at the industrial scale, preliminary steps illustrating the potential ability to grow electrically or magnetically interesting films conformally on three-dimensional substrates should be considered. For that purpose, the films were deposited on a 3D stacked silicon surface with an aspect ratio of 1:20 (Figure 4). It can be seen in Figure 4 that the film is indeed uniform on the 3D surface and it is also important to demonstrate that atomic layer deposition provides a solution for how to deposit the film inside the pattern, without covering the top. The film surface scans on planar substrates indicated certain changes in the morphology, likely related to crystal growth and roughening of the surface already in as-deposited state (Figure 4).

**Figure 5:**
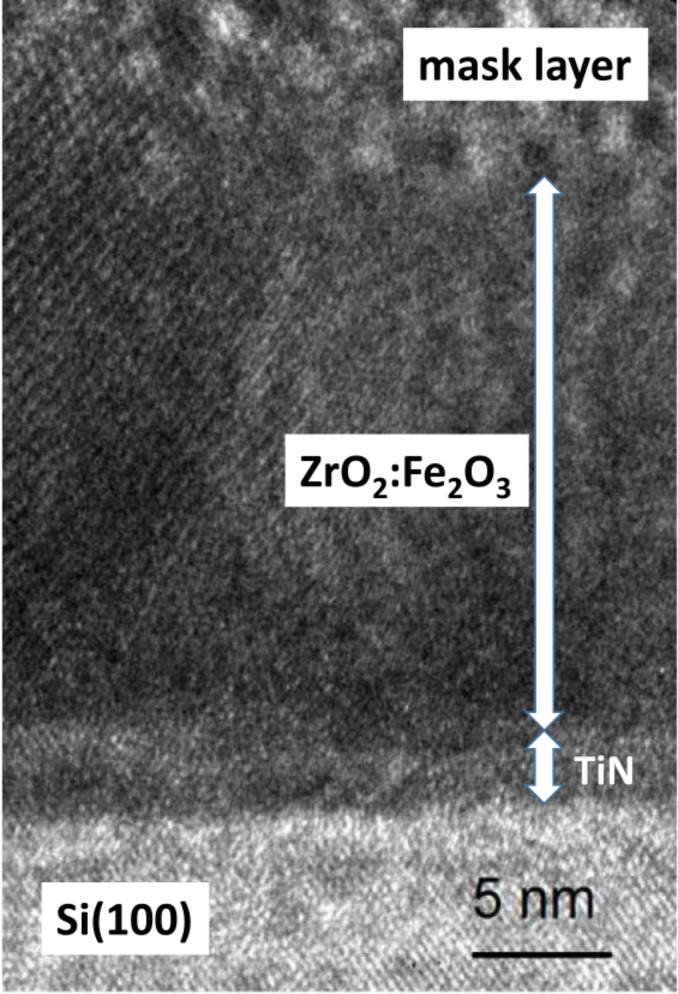
TEM image of a ZrO_2_/Fe_2_O_3_ sample with cycle ratio 10:3 on TiN. 231 atomic layer deposition cycles were deposited.

Constituent layers of the sample can be seen in the transmission electron microscope image (Figure 5). The interface between the oxide film and TiN electrode layer is distinguishable; the film is dense and the crystal growth has started immediately together with the nucleation process. The TEM image proves the formation of a stacked layer structure.

### Electrical and magnetic properties

**Figure 6:**
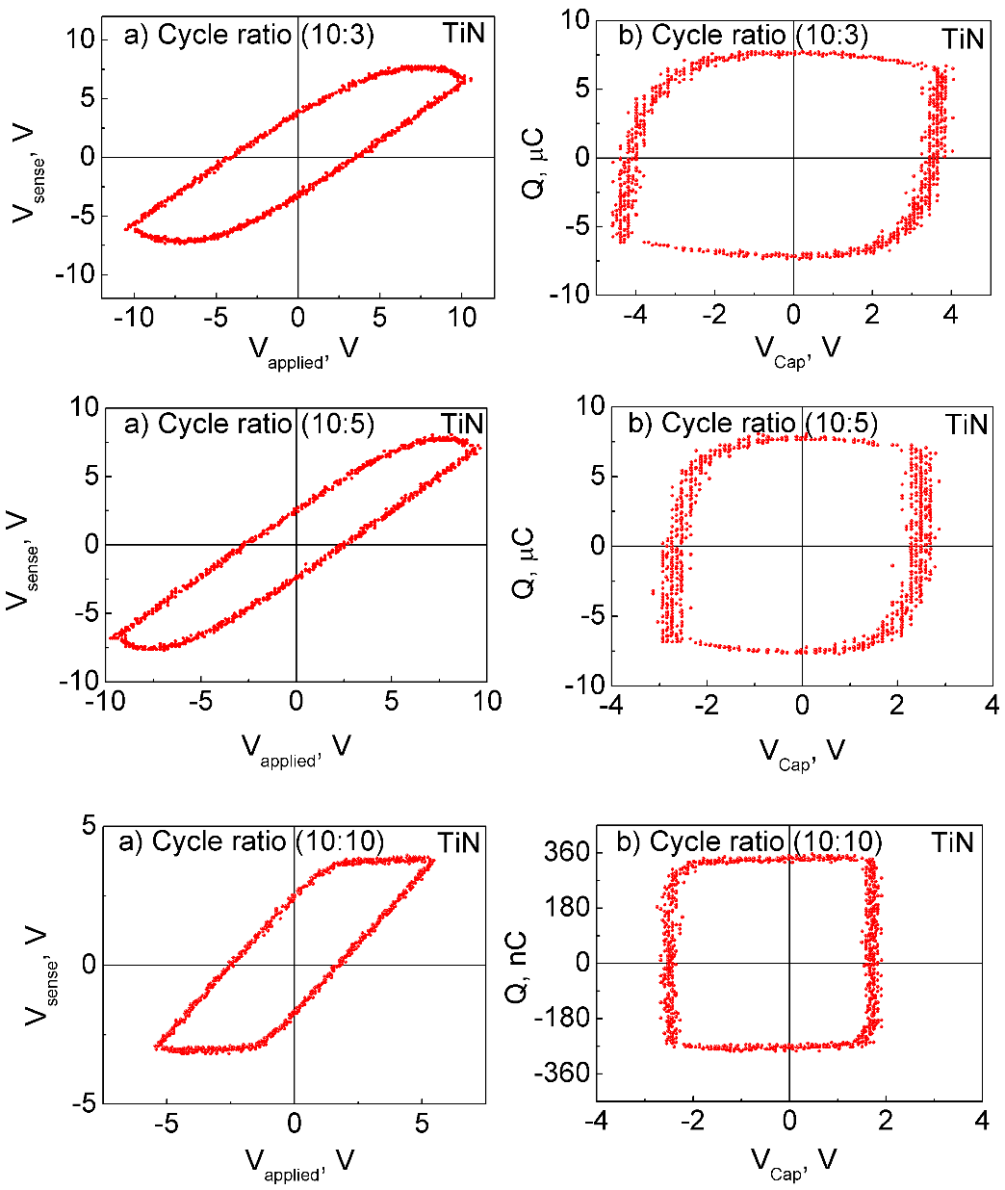
Each of the three panels show a sensed voltage–applied voltage curve on the left (a) and a polarization charge–applied voltage curve on the right (b) for Pt/ZrO_2_/Fe_2_O_3_/TiN/Ti/Si(100)/Al samples. ZrO_2_/Fe_2_O_3_ cycle ratios are shown on each image.

Most samples exhibited charge polarization, as shown in Figure 6. Charge polarization was found to decrease as the amount of Fe_2_O_3_ in the films increased (Figure 6). The charge polarization–applied voltage loops, in principle, look similar to those observed earlier for materials grown by ALD and targeted as ferroelectrics, such as LiNbO_3_ [36–37], Bi_4_Ti_3_O_12_ [38], or Pb(Zr,Ti)O*_x_* [39]. However, these loops are not yet to be attributed to the properties of a purely ferroelectric material, but an overwhelming contribution from the interfacial polarization should be taken into account. On the other hand, for comparison, in the case of ALD-grown non-centrosymmetric orthorhombic phase of HfO_2_ stabilized by doping with foreign cations [40], well-defined ferroelectric hysteresis was recorded.

For stabilized hafnia [40] the electrical polarization charge clearly tended to saturate upon the incremental increase in the external electric field strength. Along with the backward sweep of the applied voltage towards zero field, the polarization in the material initially tended to retain a value close to the saturation level, then decreased more rapidly to a certain finite level at zero field, allowing one to consider that there exists a remnant polarization value, characteristic of classical ferroelectric behavior. Furthermore, the application of a minimum external field in the opposite direction was required to draw the internal polarization to zero, allowing one to account for a coercive force [40].

**Figure 7:**
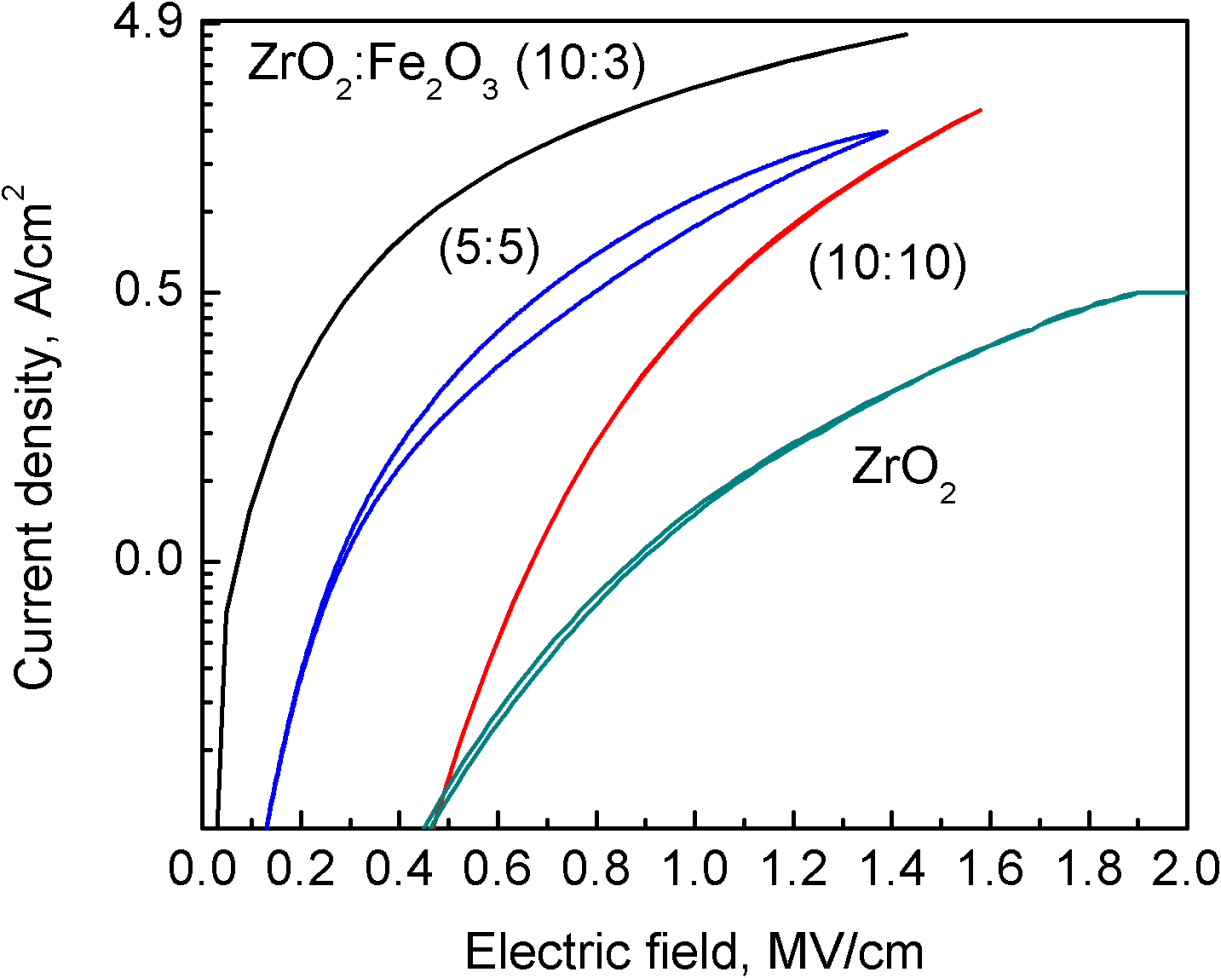
Current density–applied electric field curves for samples with ZrO_2_/Fe_2_O_3_ cycle ratios shown in the image.

In the doped ZrO_2_ films grown in the present study, any saturation level for polarization was actually not quite achieved at either polarity of the external field, as the polarized charge kept increasing with the voltage. Most probably, the charge (which is responsible for the polarization in the material deposited) was due to the electric field that drifted from an electrode to the counterelectrode. That charge, either electronic or ionic, may become trapped at the interface between the metal oxide layer and the electrode, giving rise to the interfacial polarization. It must be considered that in the vicinity of an interface a material is always most prone to defects and its lattice the most open. This is due to the usual mismatch between the crystal structure of the substrate and functional layer. The 10 nm thick TiN electrode layers were nanocrystalline, without preferred orientation, demonstrating weak and broad 111 and 200 reflections (Figure 2). ZrO_2_ and Fe_2_O_3_ phases do not possess lattice structure, allowing commensurate growth on TiN. The epitaxial relationship between magnetite (Fe_3_O_4_) and titanium nitride might be considered [41], but magnetite was not recognized in this study in the XRD patterns and, most importantly, the very first layers deposited in contact with the electrodes were always zirconium oxide. The zirconium oxide component in the films is evidently, as revealed by the X-ray diffraction studies, crystallized in the form of a tetragonal/cubic polymorph, which, in turn, is indicative of the defective nature of the material. The majority of defects may arise from the oxygen deficiency, which may be considered as one of the most influential factor stabilizing the tetragonal/cubic polymorph [42] in addition to the impurities deforming the lattice. Consequently, the electrical charge becomes carried to and trapped at the interface layer under certain polarity, and an opposite polarity with increasing, oppositely directed field is required to release the charge from the traps for the subsequent drift towards the counterelectrode. The current density to applied electric field curves are shown in Figure 7 to support the given explanation. The charge polarization values of the samples correlate with the respective leakage current through samples. The leakage currents in the film grown with ZrO_2_/Fe_2_O_3_ cycle ratio 10:5 (not shown) were similar to those in the film grown with cycle ratio 10:3, which were considerably higher than the leakage currents through other samples. Also, the charge polarization values were the highest for the samples grown with ZrO_2_/Fe_2_O_3_ cycle ratios 10:3 and 10:5 (Figure 6). Other mixtures possessed both lower leakage currents and considerably lower charge polarization values. Probably, in the mixture of iron and zirconium oxides, the amount of defects (in particular oxygen vacancies) is increased due to the substitutive exchange between metal ions of different valence, resulting in an increase also in the leakage currents. Pure ZrO_2_ exhibited the lowest leakage current (Figure 7) and did not show any significant charge polarization at all.

**Figure 8:**
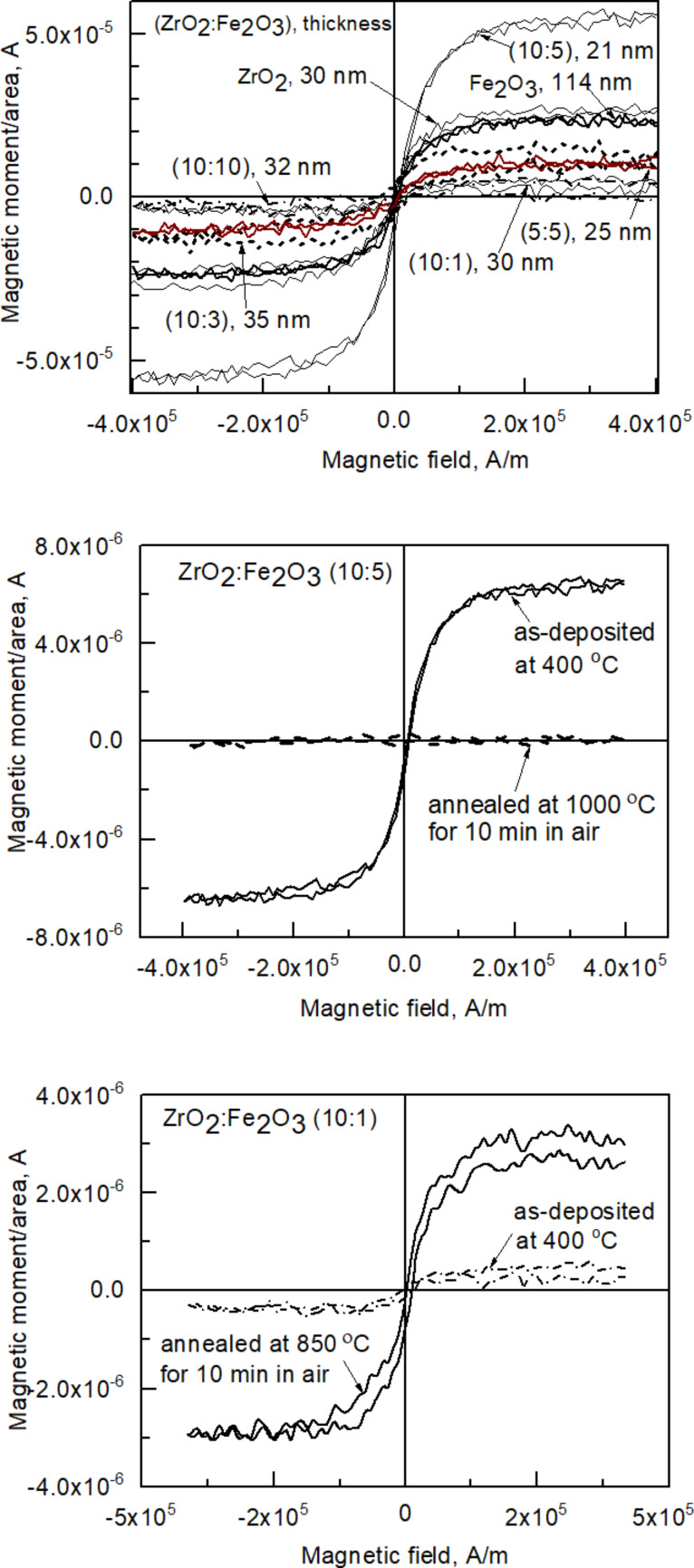
Selected room temperature magnetization–field curves for ZrO_2_/Fe_2_O_3_ films. Cycle ratios and thicknesses are indicated in the labels.

However, most samples in the present study exhibited ferromagnetic-like behavior at room temperature as shown in Figure 8. Saturation magnetization, reaching M/S = 5 × 10^−6^ A as the maximum value obtained per unit area, could be observed in most samples, but no definite hysteresis was determined in any case. The ZrO_2_/Fe_2_O_3_ sample with cycle ratio 10:5 (with cation ratio Zr/Fe = 2.0) showed considerably higher saturation magnetization with the value very similar to that measured by Sangalli et al. [19]. It is also worth noting that pure ZrO_2_ exhibited higher saturation magnetization than other mixed samples. In the case of a cycle ratio of 10:1 and Zr/Fe = 50, annealing this sample at 850 °C resulted in the increase of the observed saturation magnetization. Annealing the sample with a cycle ratio 10:5 and Zr/Fe = 0.34 at 1000 °C had an opposite result and removed the saturation magnetization of the film (Figure 8).

For comparison with the literature, Myagkov et al. [5] have synthesized Fe–Fe_3_O_4_–ZrO_2_ nanocomposite ﬁlms by thermally depositing and oxidizing Fe films, followed by the sequential deposition of Zr layers and annealing of the double metal oxide–metal stacks. In this study, saturative magnetization appeared in the samples heat treated above 250 °C and was recorded up to 500 °C, above which nonferromagnetic phases supposedly started to form. In other studies by de Souza et al. [33], Kuryliszyn-Kudelska et al. [34] and Okabayashi et al. [10], only paramagnetic behavior in Fe-doped ZrO_2_ was observed. One can note that, for example, in the work by Okabayashi et al. [10], annealing was presumably required in order to initiate crystallization in sol–gel-synthesized (and therefore initially amorphous) films. Magnetization-field hysteresis loops were recorded in the ZrO_2_ films co-doped with both iron and cobalt, whereas in the films doped only with 1% iron, only paramagnetic behavior was observed [10]. In our films in the present study, the material layers were evidently crystallized already in the as-deposited state, which was sufficient for the appearance of magnetization loops, However, the annealing procedure may have resulted in the formation of nonferromagnetic phases, possibly due to the increased contribution from monoclinic ZrO_2_ due to the partial compensation of the oxygen deficiency in the films upon heat treatment in air. It is, however, worth noting that the recrystallization or transformation into monoclinic phase cannot be regarded as a fast process, because there appeared only very weak traces of the monoclinic polymorph, expressed by an almost insignificantly low additional reflection at 31.3°, otherwise being indicative of 111 reflection of monoclinic ZrO_2_ (PDF Card 37-1484). It is also worth noting that magnetization even in ZrO_2_ that is not doped with ferromagnetic metals has been investigated and theoretically predicted [43]. In another study on electron beam evaporated undoped ZrO_2_ films, ferromagnetic-like magnetization hysteresis was recorded, whereby the saturation magnetization was positively correlated with the amount of metastable tetragonal/cubic zirconia in relation to the stable monoclinic phase in the films [6].

## Conclusion

Zirconium oxide mixed with iron oxide thin films with various cycle ratios of constituent oxides were grown by ALD to thicknesses ranging from 15 to 40 nm from ZrCl_4_, Fe(C_5_H_5_)_2_ and O_3_. Most of the films exhibited charge polarization and saturation magnetization. The highest numerical values of these physical quantities were achieved in samples with a relative low iron content (Zr/Fe > 2.0). The saturation magnetization per unit area in a film with ZrO_2_/Fe_2_O_3_ cycle ratio of 10:5 was about 5 × 10^−5^ A and the charge polarization at 0 V applied voltage was about 8 µC. These samples were crystallized in the tetragonal/cubic phase of ZrO_2_. As-deposited samples, in which the relative cation content for zirconium decreased below 2.0, did not show any peaks assignable to zirconia.
